# MIGRATE OR STAY? PSYCHOLOGICAL WELL-BEING AMONG MIGRANT AND LEFT-BEHIND OLDER ADULTS IN CHINA

**DOI:** 10.1093/geroni/igad104.2171

**Published:** 2023-12-21

## Abstract

When adult children migrate from households, older parents face two options: migrate with children or remain in place (“left behind”). However, it remains unclear which option is better for older adults’ psychological well-being. With the data from China Health and Retirement Longitudinal Survey (CHARLS) at the wave of 2015 and 2018 (N = 4989), this study examined the longitudinal changes in depression and life satisfaction among older locals (those who did not migrate and lived with their children), migrants and left-behinds, and the impacts of children support and social support on the well-being of older parents. Results showed that there was an increase in depression level among the left-behind older adults from 2015 to 2018, but this was not found among the migration group or the local group. Furthermore, the left-behind group reported a higher level of depression than the local group and migration group, and this difference was accounted by the time of living with children, but not social activities. In addition, the time of living with children was positively associated with life satisfaction among the left-behind group, and this association was not observed among the migration group or the local group, suggesting the salutary effect of children’s support among the left-behind group. These results highlight the adverse effect of being left behind among older adults and the importance of children’s support for left-behind older adults.

